# Internet use predicts Chinese character spelling performance of junior high school students: multiple mediating roles of pinyin input proficiency and net-speak experience

**DOI:** 10.3389/fpsyg.2023.1153763

**Published:** 2023-08-11

**Authors:** Rong Luo, Yifan Peng, Jingjun Chen

**Affiliations:** Department of Psychology, Hunan University of Science and Technology, Xiangtan, China

**Keywords:** internet use, pinyin input proficiency, net-speak experience, multiple mediating, Chinese character spelling

## Abstract

To examine the complex relationship between Internet use experience and character spelling performance among Chinese junior high school students, the study explored the multiple mediating roles of Pinyin input proficiency and net-speak experience. A total of 447 Chinese junior high school students aged 12–15 years old completed the Internet Use Experience and Pinyin Input Proficiency Assessment, the Net-speak Experience Questionnaire and the Chinese Spelling Test. The results showed that: (1) All investigated variables were significantly correlated with each other, but there was no direct relationship between Internet use and Chinese spelling performance. (2) Pinyin input proficiency and net-speak experience play a chain mediating role in the relationship between Internet use and Chinese character spelling performance. Teens’ Internet use experience indirectly and positively predicted Chinese character spelling performance through the mediation of Pinyin input method use and net-speak experience. The implication of this study is that Chinese children should be guided to engage in Internet activities that require Pinyin typing and use net-speak creatively in order to promote the traditional Chinese character spelling skills when instructing teenagers to engage in Internet activities.

## Introduction

According to *The 48th China Statistical Report on Internet Development* released by China Internet Network Information Center, as of June 2021, the number of Internet users in China reached 1.011 billion, the number of mobile phone users reached 1.007 billion, and the number of instant message users reached 983 million, accounting for 97.3% of the total number of Internet users; the proportion of teenager Internet users using mobile phones reaches 90%, among which the number of Internet users aged 6 to 19 reached 185 million, accounting for 15.7% of the total number of Internet users, with the largest group of students of middle school, high school and other secondary education levels. It can be seen that, with the rapid development of modern information processing technology and network communication technology, the number of young Internet users is on the rise. With the popularization of the Internet, a series of changes have taken place in the way of reading and writing among young people. In terms of reading, paper materials are gradually being replaced by electronic documents (or web pages); in terms of writing, handwriting is being replaced by keyboard input, and digital writing (such as Blog etc.) is becoming increasingly popular; in terms of information communication, Internet expression and communication (such as WeChat, QQ, etc.) is becoming widespread. As most of these activities are related to language activities (communication, reading, writing, etc.), does the change in the medium of reading and writing have an impact on the psychological processes of reading and writing, and therefore on children’s traditional literacy development?

So far, a large number of studies have focused on the relationship between Internet use and reading, but few studies have focused on the relationship between Internet use and Chinese character spelling (Xu et al., 2020; Chen et al., 2021). For Chinese children, the relationship between Internet use and writing is complicated. According to *the 2020 National Research Report on the Internet Use of Minors*, 82.9% of Chinese underage Internet users have their own Internet access devices, with mobile phones being the most popular electronic devices. The data showed that the proportions of junior high school students studying online, chatting and using social networking sites are 90.7, 74.2 and 45.7% respectively, which are higher than the overall level of Internet users. When Chinese children engage in Internet activities, they will use Pinyin input method to type Chinese characters when it comes to doing online homework, sending messages, etc., which is completely different from English typing. Studies (Xu et al., 2020; Chen et al., 2021) show that, the use of Pinyin input method is beneficial to Chinese character spelling. In addition, Chinese children can also use net-speak in Internet communication. However, the form of Chinese net-speak is completely different from that of English. The latter is more of an abbreviated form of standard English, while the former is more of codified characters or Chinese standard characters that extend new meanings on the Internet. That is, old words are endowed with new implications (Chen et al., 2020). Studies have also shown that the use of net-speak seems to be beneficial to Chinese character processing (Chen et al., 2020), which implies that the use of Pinyin input method and net-speak style in Internet activities by Chinese children may be beneficial to their mastery of standardized Chinese character spelling.

A survey has shown that 97% of primary and middle school students use Pinyin input method in their Internet activities (Chen et al., 2016). Another survey also found that middle school students are most familiar with net-speak (Jin and Cao, 2010). Will the extensive use of Pinyin input and the familiarity of net-speak have a beneficial effect on the Chinese character spelling of middle school students? In addition, is it true that the more children use the Internet, the more proficient they are in Pinyin input and the more familiar they are with the net-speak, which in turn positively affects their standard writing level? This study hypothesizes a multiple mediation model to answer the above questions.

## Literature review

### Internet use and literacy

The Internet is the world’s largest open network of computers of all sizes connected to each other. The Internet plays a different role in the learning lives of young people, and its emergence has made a huge difference to the environment in which they grow up. Recent estimates show that one third of the world’s young people under the age of 18 use the Internet, and in developed countries, 75% of young people play video games every day. The latest results from the US ESA show that young people spend more than 11 h a day on modern electronic media such as computers, the internet and video games, with 71% of young people under the age of 18 playing video games regularly. The under-18 group accounts for 24% of video game players (ESA, 2022).

Teenagers’ internet use has a serious impact on their mental health, social, educational and emotional development (Kirmayer et al., 2013). Surveys have shown that the growing incidence of teenagers still being addicted to the Internet and that excessive Internet use makes teenagers depressed, anxious (Cataldo et al., 2021; Saadati et al., 2021; Lozano-Blasco et al., 2022). A range of mental problems such as loneliness and corresponding interpersonal troubles (Hao et al., 2022). However, as a new learning tool, information medium and cultural carrier, among other things, most young people are still currently profiting reasonably well from the Internet (de Sousa et al., 2022). Internet use has led to improved memory and attention span and more active thinking skills among teenagers (Casares and Ramirez, 2019). In addition, early experiences with online technologies may have a beneficial impact on children’s later academic performance and digital skills as well as on their cognitive development (Hurwitz and Schmitt, 2020).

In terms of the relationship between Internet use and teenager literacy, researchers have focused more on the relationship between Internet use and reading, writing and spelling, and in general, the researches that have been conducted showed mixed results.

### Internet use and reading

In terms of reading, researchers (Umbara et al., 2015) found a significant correlation between Internet use and reading comprehension levels (*r* = 0.2). Petko et al. (2017) found that using ICT to finish homework (browsing the Internet to finish homework, using email to communicate with other students about homework, and doing homework on the computer) was positively associated with reading scores. Consistent with this finding, it was found that students who used ICT resources more frequently for leisure activities (e.g., playing online games, chatting online, reading news on the Internet) tended to perform better on reading tests (Hu et al., 2018). A survey on Mexican university students (Márquez et al., 2020) also found that students who used the Internet frequently not only read more digital texts, but also paper materials. Whereas a study on university students in Thailand (Anthoney et al., 2020) found no significant relationship between Internet use and reading attitudes, some studies have found negative effects: Casey et al. (2012) found that instant message was negatively associated with reading performance. In recent years, researchers (Borgonovi and Pokropek, 2021) have analyzed the relationship between Internet use and reading performance among students in 28 countries and found an inverted U-shaped association between different levels of Internet use and reading performance: students with low and high levels of Internet use have lower reading performance than students with moderate levels of Internet use. Overall, there was a positive effect of appropriate levels of Internet use on students’ reading performance.

### Internet use and writing

In terms of writing, researches have focused more on the impact of ICT use on writing. A study by the US Internet Research Center (Lenhart et al., 2008) on the relationship between teens’ technology use and writing noted that although children often used the Internet to write, they did not consider email, instant messaging and short messages to be writing. In addition, both children and their parents felt that the use of technology had both positive and negative effects on writing. The positive one is that it was easier to write better because they can revise and edit easily, present ideas clearly, communicate well and they tend to be more creative. The negative effects include taking short cuts and not putting effort into writing, using wrong spelling and grammar, writing too fast and being careless, having a short attention span. Weiser et al. (2019) found that the reading, writing and spelling performance of students with reading learning disabilities was improved by giving teachers technology support. Among the technology supports were how to record and upload lessons and search for resources on the platform that would be beneficial to both teacher instruction and student engagement and learning. Fernández Batanero et al. (2021) reviewed literature from 2010 to 2020 that examined the use of ICT in writing and reading and found that although science and technology in this area was still underdeveloped, digital technology may contribute to the development of literacy skills.

### Internet use and spelling

In terms of spelling, as digital writing is mostly to be found in children’s Internet communication activities, the previous studies have focused more on the effects of using information communication (e.g., text message and instant message) in Internet use, with related studies showing inconsistent results. For example, some studies have shown no significant relationship between the frequency of Internet information technology-mediated communication and traditional spelling skills (Verheijen, 2013). Similarly, Zebroff and Kaufman (2017) also indicated that text messaging practices did not appear to be significantly associated with spelling among teenagers aged 15–17. While Vella (2014) found a negative effect of chatting on spelling performance among Maltese Secondary School students, generally the higher the frequency of chatting is, the lower the literacy score is. Chen et al. (2021) noted that, if just considering the frequency or time of IM/texting, there was a significant negative correlation between literacy skills and IM/texting. However, the use of textism, an abbreviated language style, in online communication appears to improve literacy skills, as Plester et al. (2009) found the positive correlation between the use of text message abbreviations and spelling ability among 88 British 10-12-year-old children. A study by Kemp and Bushnell (2011) of 86 Year 5–6 children in Australia also showed that text-messaging practices improved spelling skills. Mills (2014) also indicated that texting could be a literacy practice based on instructional purpose. In conclusion, the use of the Internet seems to be an “oxymoron” for English spelling, with the negative effects of excessive use on the one hand, and the positive effects of textism in the process on the other. This may be one of the main reasons why some studies have not found a direct relationship between Internet use and English spelling, and it is likely that Internet use is only a distal variable affecting spelling levels, while frequency of IM or texting and language style are intermediate variables.

### Relationship between pinyin input, net-speak, and Chinese character spelling

Chinese Pinyin is a tool to assist in the pronunciation of Chinese characters. It is the official Latinization scheme for the pronunciation of Chinese characters promulgated by the People’s Republic of China and is mainly used for the pronunciation of Mandarin Chinese characters as a kind of Mandarin phonetic symbol. Input method is a way of encoding various language symbols into a computer, mobile phone or other device, and is a writing tool that has been created by the development of human writing, breaking the limits of ink. Unlike English, which has the advantage of 26 Latin letters, Chinese characters are a combination of phonetics, morphology and semantics, and are basically typed using a combination of phonetics, morphology and semantics linked to specific keys, and then combined according to different Chinese characters. In other words, the Pinyin input method is used to input Chinese characters according to the Pinyin regulations.

Handwritten Chinese characters evolve from semantic activation, then orthography and speech automatically, and then glyphs are produced by means of the Peripheral Motor Process. Therefore, children need both phonetics and written morphology to write Chinese characters. Pinyin input involves both the process of speech production and the recognition of Chinese characters (Chen et al., 2017) by first typing Pinyin based on semantics and then selecting the desired character from alternative homophones. Numerous studies have found that pinyin input experience facilitates both phonological and orthographic processing, and that high proficiency in Pinyin input is beneficial for character recognition and further improves literacy performance. Zhu et al. (2009) found that Pinyin input experience facilitated phonological and orthographic processing through a study on the relationship between Pinyin input and literacy performance among university students, indicating that Pinyin input proficiency is positively related to individual literacy ability. Chen et al. (2016) invited primary school students in grade 6 to separately use two methods (handwriting and Pinyin input) to learn new characters and review newly-learned characters, and found no significant differences in the effects of the two methods. Further findings indicated that Pinyin input experience indeed strengthened the link between semantics and phonology, and did not weaken the link between semantics and orthography. Xu et al. (2020) found that junior high school students’ use of Pinyin as a digital writing tool during Internet use could “shelter” its direct negative effect on Chinese character handwriting ability through the positive mediation of Pinyin input proficiency. Chen et al. (2021) also found that Pinyin input experience played a positive mediating role in the prediction of handwriting Chinese character performance by instant messaging (IM). This suggests that the use of Pinyin input method is beneficial to teens’ Chinese character writing. The reason for this, according to the researcher (Chen et al., 2017), is that pinyin input involves first typing Pinyin based on semantics and then selecting the required Chinese character from alternative homophones, whereby the Pinyin input process involves the process of speech production as well as the process of Chinese character recognition, which means that it reinforces both Chinese phonology and orthography.

In terms of teens’ net-speak usage style, English net-speak is mostly used in abbreviated form, called textism (Chen et al., 2020). As textisms are informal (unconventional spelling and grammatical shortcuts) and differ significantly from standardized English words or phrases in terms of orthography and spelling rules, there is concern that the long-term use of textisms may have a negative impact on children’s normative literacy skills. A number of studies have examined the relationship between children’s use of textisms and normative literacy, but most of them have demonstrated that children who use textisms more often score higher on literacy-related assessments (Waldron et al., 2015; Blom et al., 2017); these literacy skills include: word reading (Plester et al., 2009; Coe and Oakhill, 2011), phonological awareness (Plester et al., 2009; Wood et al., 2014) and spelling (Plester et al., 2009; Bushnell et al., 2011; Wood et al., 2014). Most textisms appear to be homophonous variant of standard English vocabularies, which implies that, for net-speak experienced speakers, English net-speak is closely related to standard words in the individual’s psychological vocabularies, so that high proficiency in English net-speak implies high proficiency in standard English. For Chinese net-speak, most of its forms are homographs, and the study (Chen et al., 2020) also found that the more experienced college students are in net-speak, the better their recognition and judgment of normative vocabulary are. In summary, studies using both English and Chinese as materials suggest that net-speak experience can benefit teens’ literacy skills.

Internet chatting activities ranked second in teens’ Internet use in China (CNNIC, 2021), which means that teens have a lot of opportunities to use net-speak in Internet communication, and as researchers have pointed out, most teens are not satisfied with normative words and use net-speak more frequently in their Internet communication (Grace et al., 2012; Drouin and Driver, 2014; Wood et al., 2014). Clearly, the more frequently the Internet is used and the more experienced the teens are in using it, especially with the frequent use of communication mediated by Internet information technology, the more experienced they are in net-speak. For Chinese teens, Chinese characters are the only ideographic system in the world that is still widely used and cannot be typed directly on a computer keyboard, and 90% of Chinese teens use Pinyin input methods to input Chinese characters in various Internet activities (Chen et al., 2021). Children overwhelmingly use Pinyin input method for both standardized Chinese words and Chinese net-speak. It is reasonable to believe that the more children use Pinyin input method in online communication, the more familiar they will become with net-speak and the more experienced they will be with net-speak.

### Present study

The research objectives of this paper: Firstly, to identify the mechanisms by which teenagers’ Internet use affects the traditional literacy skills. Secondly, to explore novel pathways for teenagers’ use of the Internet for Chinese character learning.

Given that previous studies have suggested that the relationship between Internet use and English spelling seems to depend on certain intermediate variables (e.g., text message frequency or net-speak style), that Internet chatting is the most widespread non-educationally relevant activity for Chinese children, and that the study on the relationship between Internet use and character spelling among Chinese children is insufficient in the previous literature, this study hypothesizes, based on the previous analyzes, that Internet use positively affects character spelling performance through Pinyin input and net-speak experience. The present study hypothesized multiple mediating mechanisms through which Internet use positively affects spelling performance through Pinyin input and net-speak experience. A survey method was used to collect data on junior high school students’ Internet use, Pinyin input proficiency, net-speak experience and to test word spelling performance. Then a multi-mediation model was established to test the above hypothesis.

## Method

### Participants

Four hundred fifty-two teens aged between 12 and 15 were selected as participants by cluster sampling. Data were collected from 3 different junior middle schools in Hunan Province of China. 49.1% (222) are males and 49.8% (225) are females (gender data of 5 participants are missing). The numbers of students from Grade 7 to 9, account for 33.6, 31.2, and 35.2%, respectively, (shown in Table 1).

**Table 1:** Demographic profile of respondents (*n* = 447).

Variable	Measure	Frequency	Percentage (%)
Gender	Male	222	49.1
	Female	225	49.8
Grade	Seven	150	33.6
	Eight	141	31.2
	Nine	156	35.2

## Instruments

### Internet use experience

Two self-assessment questions were used to investigate teens’ experience of using the Internet. One was “How long have you been online?” with five options ranging from 0 to 1 year, 1–4 years, 5–8 years, 9–12 years, and more than 13 years; the second was “If you were asked to rate how often you usually go online, how often do you think you do?,” with options ranging from “rarely” to “very frequently” on a 5-point scale (shown in Table 2).

**Table 2:** Key variables and questionnaire items.

Variables	Source	Items	Measures
Internet use experience	Chen et al. (2020)	“How long have you been online?” “If you were asked to rate how often you usually go online, how often do you think you do?”	0–1 year, 1–4 years, 5–8 years, 9–12 years, and more than 13 years; “rarely” to “very frequently” on a 5-point scale.
Pinyin input proficiency	Chen et al. (2020)	if the maximum score of proficiency and ability about using Pinyin method to input Chinese character is rated 10 points, what is your assessment of yourself?	Pinyin input level on a 10-point scale from 1 to 10.
The net-speak experience questionnaire	Chen et al. (2021)	A selection of 23 internet language words were presented, such as “hold住”	Six options ranging from “I have never heard of it” to “very frequently used.”
Word spelling test	Chen et al. (2021)	In each word, one word was missing, “kè zhàn 客 ()”; in a sentence, the word to be spelt was missing, “His face yáng yì () had a heartfelt smile on it.”	The scoring system is one-word-one-point. The correct answers for the two examples are: “kè zhàn 客 (**栈**)"and, “His face yáng yì (**洋溢**) had a heartfelt smile on it”

### Pinyin input proficiency

Teens needed to self-assess their proficiency in Pinyin input (Chen et al., 2020). The question was: if the maximum score of proficiency and ability about using Pinyin method to input Chinese character is rated 10 points, what is your assessment of yourself? Participants needed to rate their Pinyin input level on a 10-point scale from 1 to 10 (shown in Table 2).

### The net-speak experience questionnaire

The Net-Speak Experience Questionnaire (See Supplementary materials) developed by Chen et al. (2021) was used. The 50 net-speak words used in the questionnaire came from http://wangci.net/and are currently the most commonly used and most popular Chinese words. After an exploratory factor analysis following the initial test, 23 net-speak words were screened as material for the formal survey. For each word, a six-point rating scale was adopted and the participants were asked to choose from six options ranging from “I have never heard of it” to “very frequently used.” The Cronbach’s α of the questionnaire was 0.967 (shown in Table 2).

### Word spelling test

Forty Chinese characters and 30 words marked with the correct Pinyin are used to test writing levels (See Supplementary materials). In every two-character word, one character was missing and participants were asked to fill in the blank such as “kè zhàn 客 ()”. Words are presented in sentences. In a complete sentence, the word to be spelt was missing, e.g., “他的脸上 yáng yì () 着会心的笑容.” The scoring system is one-word-one-point. The correlation coefficient was *r* = 0.453, *p* < 0.001, using the teens’ language scores as a calibration standard for examining word writing tests. It means that the criterion validity of writing Chinese character test is sufficient (shown in Table 2).

## Results

### Gender and grade differences

Gender and grade level may influence Internet use and Chinese character spelling performance. Boys and girls have different preferences for Internet use, with girls communicating more frequently online (Zhou, 2014; Zhu et al., 2017). Girls often express intimacy through “words,” while boys build friendships through non-verbal means such as “games,” so girls’ use of social services on the Internet is more prominent (Lei and Liu, 2005). These findings seem to suggest that girls have more access to keyboarding and may perform better in Chinese character spelling. Lei and Liu (2005) surveyed 339 students in middle school and found that adolescent students’ preference for Internet social services increased with grade level, with a qualitative change in adolescents’ preference for Internet social services occurring in the Grade 2 of junior middle school and continuing to increase thereafter. Grade level reflects age, with the higher the grade level, the longer the exposure to the Internet, the more experience with digital writing activities, and the more practice with literacy skills.

MANOVA (multivariate analysis of variance) was adopted to analyze the effects of gender and grade on individual variables. The means and standard deviations are shown in Table 3.

**Table 3:** The means and standard deviations of various factors by gender and grade (*M*, SD).

	*M* (SD)		*M* (SD)		*M* (SD)	
Total *n* = 44	Grade 7		Grade 8		Grade 9	
	Male *n* = 67	Female *n* = 83	Male *n* = 72	Female *n* = 69	Male *n* = 83	Female *n* = 73
Internet use experience	4.75 (1.69)	3.99 (1.61)	5.19 (1.71)	4.07 (1.59)	5.59 (4.31)	4.23 (1.63)
Spelling performance	26.07 (16.25)	28.93 (15.37)	27.70 (17.60)	34.91 (15.04)	33.42 (17.25)	41.49 (14.85)
Pinyin input proficiency	5.73 (2.00)	5.81 (2.11)	6.42 (2.57)	6.52 (1.86)	6.38 (2.06)	6.66 (1.94)
Net-speak Experience	78.89 (24.93)	70.19 (25.30)	82.05 (25.67)	77.44 (21.70)	79.35 (23.39)	84.05 (24.14)

The results of the multivariate ANOVA showed significant main effects for gender (*Wilks’ Λ* = 0.919, *F* = 9.70, *p* < 0.001, *η2 p* = 0.081) and grade (*Wilks’ Λ* = 0.915, *F* = 4.95, *p* < 0.001, *η2 p* = 0.043), but both the interaction effects were not significant. Gender differences in Internet use (*F* = 17.41, *p* < 0.001, *η2 p* = 0.038) and Chinese character spelling performance (*F* = 15.64, *p* < 0.001, *η2 p* = 0.034) were highly significant, with boys using the Internet significantly more than girls and girls outperforming boys in Chinese character spelling. Grade differences were also significant for Internet use (*F* = 3.89, *p* = 0.021, *η2 p* = 0.017) and Chinese character spelling (*F* = 14.80, *p* < 0.001, *η2 p* = 0.063), with an increasing trend from first to third year. We also found no significant gender differences in children’s ratings of Pinyin input proficiency and Net-speak experience, but there were significant grade differences (*F* = 5.89, *p* = 0.003, *η2 p* = 0.026；*F* = 3.06, *p* = 0.048, *η2 p* = 0.014), which also showed an increasing trend with grade.

### Partial correlation among internet use, pinyin input proficiency, net-speak experience and spelling performance

Based on the above results, we adopted partial correlation to examine the relationships among variables by controlling gender and grade. The results are shown in Table 4.

**Table 4:** Partial correlation coefficients of variables among junior middle school students (*r*).

Variable	1	2	3	4
Internet experience	1			
Spelling performance	0.12**	1		
Pinyin input proficiency	0.23***	0.23***	1	
Net-speak Familiarity	0.34***	0.22***	0.31***	1

*N* = 447, **p = 0.012, ***p < 0.001.

The results showed that all investigated variables were significantly correlated with each other, satisfying the prerequisites for a mediation analysis. In conjunction with the aforementioned hypothesis of a mediating process between Internet use and Chinese character spelling performance, a multiple mediated path model was developed and validated in this study.

### A path model of internet use predicting spelling performance

**Figure 1:**
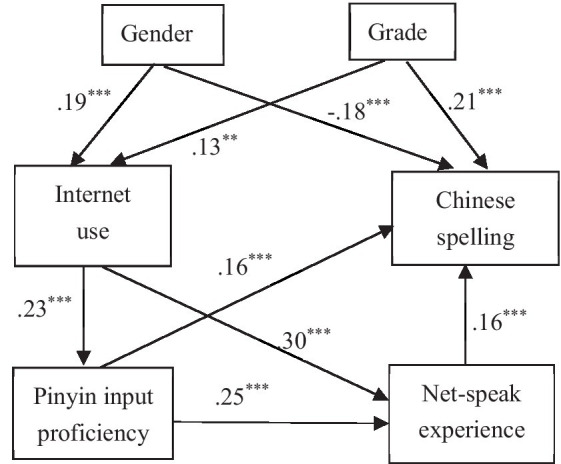
Path model of the relationships among variables (standardized path coefficient; **p < 0.01, ***p < 0.001. Gender is dummy variable. Female is 0, male is 1).

Amos software was used to fit for the path model and the model of the path analysis is shown in Figure 1.

The results of path analysis show that Internet experience has an indirect positive effect on the Chinese character spelling. Bootstrap was used to test the significance of the mediate effect. The number of bootstrap samples is 2000. The results show that standardized indirect effect is.098 and bias-corrected 95% confidence interval is [0.053, 0.157] which does not include 0. It means that the mediate effect of Pinyin input proficiency and net-speak experience is significant between teens’ Internet use and Chinese character spelling performance. The fitting indexes are shown in Table 5.

**Table 5:** Fitting indexes of path model.

*χ*^2^	df	*χ*^2^/df	GFI	CFI	NFI	IFI	RMSEA	SRMR
12.345	6	2.058	0.991	0.971	0.947	0.972	0.048	0.038

Generally speaking, GFI, CFI, NFI and IFI should be higher than 0.9. RMSEA and SRMR should be lower than 0.05. The results of fitting indexes show that the path model is acceptable.

## Discussion

The study found that teens’ Internet use indirectly, significantly and positively predicted Chinese character spelling performance through the mediation of Pinyin input and net-speak experience. There was no direct relationship between Internet use and Chinese character spelling performance, which is consistent with the findings of some studies using English as materials (Verheijen, 2013; Zebroff and Kaufman, 2017). Furthermore, this finding demonstrates that Internet use is a distal variable predicting Chinese character spelling performance, acting on writing through Pinyin input and net-speak experience.

For 90% of Chinese children, Pinyin input method is used whenever Chinese character typing is involved in the use of the Internet and its ancillary devices, whether it is for writing, web searching, email, or chatting (Chen et al., 2020). Unlike English input where letters are typed directly on the keyboard, Chinese character cannot be typed directly in the character form, but need to be typed in the Pinyin form of the character, which matched the character form by the built-in character database of the input method software, and then the correct character is selected from a number of homophones and anagrams. For example, if the pinyin for“汉字” is “hàn zì” and you want to input the word “汉字,” you can type the letters “h-a-n-z-i” in order, the input method software may show“汉子”“汉字”“憨子”“汗渍” and other options. In this process, the typist activates both the phonetic sound of the Chinese character and receives feedback reinforcement of the character’s form. Experimental studies have shown (Chen et al., 2017) that Pinyin input experience indeed strengthened the link between semantic and phonology, and did not weaken the link between semantics and orthography. Learning new Chinese characters by using the Pinyin input method likewise contributed to the mastery of Chinese handwriting (Chen et al., 2016). The present study also confirms that Pinyin input experience facilitates the spelling of Chinese characters.

Chatting is one of the most popular online activities for children in China. The net-speak (texism) used by children in chatting often has a different style from standard language. English net-speak is generally abbreviated, using different forms of orthographic or phonological abbreviations (Waldron et al., 2015; Blom et al., 2017; Chen et al., 2020), such as contractions (e.g., msg for message), phonological abbreviations (e.g., thru for through), initialisms (e.g., omg for oh my God), shortenings (e.g., goin for going), single letters (e.g., u for you), combined letters (e.g., 2 day for today), and accent stylizations (e.g., gonna for going to). Plester et al. (2009) have pointed out that people experienced in net-speak themselves may have a high level of language sensitivity and ability, which is the reason why they are interested in net-speak words and use them creatively. In contrast, the form of Chinese net-speak is generally homograph, where traditional words take on new semantic meanings in the Internet context (e.g., a waistcoat originally meant a coat, but its extension in the Internet context means ID on the Internet). Proficiency in the use of net-speak is precisely based on proficiency in the standard language, and in turn, the use of net-speak reinforces the mastery of its standardized form. Research has found that Chinese net-speak experience facilitates the recognition and processing of Chinese standard words (Chen et al., 2020). In summary, studies of both Chinese and English language materials have found that the more frequently the net-speak is used, the more beneficial it is for the recognition of standard words (Plester et al., 2009; Kemp and Bushnell, 2011; Blom et al., 2017). The present study also confirms this finding.

In addition, this study also confirms that Internet use predicts Pinyin input proficiency and net-speak experience. This suggests that the more Chinese children use the Internet, the more proficient they become in Pinyin input, due to the fact that Pinyin input is a common typing software in children’s use of the Internet. Therefore, the more frequently the Internet is used, the more proficient teenagers are in using Pinyin input. In turn, most of the activities that teenagers do when using Pinyin input are online chatting, which may be the reason why Pinyin input predicts net-speak experience.

## Conclusion and implications

To sum up, this study found no direct relationship between Chinese teens’ Internet use and Chinese character spelling, but rather indirectly predicted Chinese character spelling performance through Pinyin input proficiency and net-speak experience. This implies that for Chinese children, Internet use that requires the use of Pinyin input is helpful in mastering Chinese characters, and the use of net-speak in online social interaction is beneficial for Chinese character spelling. However, this does not mean that online chatting is helpful for spelling, and research has found that without the compensatory effect of Pinyin typing, the frequency of chatting activities such as IM is actually detrimental to Chinese character handwriting (Chen et al., 2021).

This suggests that when guiding young people to engage in online activities, if the aim is to promote their Chinese spelling skills, we need to encourage them to engage in some activities that require Pinyin typing, such as computer homework, writing blogs, emails, forum comments, etc., rather than some unrelated activities such as surfing, playing games, watching videos, online shopping and other game-like activities. As for common chatting activities, on the one hand we need to limit their excessive frequency, and on the other hand, to promote text input chatting rather than voice chatting, and finally encourage creative use of net-speak in chatting.

## Data availability statement

The raw data supporting the conclusions of this article will be made available by the authors, without undue reservation.

## Ethics statement

The studies involving human participants were reviewed and approved by School of Education, Hunan University of Science and Technology. Written informed consent to participate in this study was provided by the participants’ legal guardian/next of kin.

## Author contributions

RL and JC wrote the first draft of the manuscript. YP performed material preparation and collection and analysis of the data. JC designed the study. All authors contributed to the article and approved the submitted version.

## Funding

This work was supported by the MOE (Ministry of Education) in China Project of Humanity and Social Science under Grant Number 16YJCZH067.

## Conflict of interest

The authors declare that the research was conducted in the absence of any commercial or financial relationships that could be construed as a potential conflict of interest.

## Publisher’s note

All claims expressed in this article are solely those of the authors and do not necessarily represent those of their affiliated organizations, or those of the publisher, the editors and the reviewers. Any product that may be evaluated in this article, or claim that may be made by its manufacturer, is not guaranteed or endorsed by the publisher.
