# Supplementary material for: Internet use predicts Chinese character spelling performance of junior high school students: multiple mediating roles of pinyin input proficiency and net-speak experience
Source: Front Psychol. 2023 Aug 11;14:1153763. doi: 10.3389/fpsyg.2023.1153763 (PMC10452878; doi:10.3389/fpsyg.2023.1153763)
Supplement: Supplementary file 1 [file Data_Sheet_1.PDF]

Supplementary materials 1：

The net-speak experience questionnaire

1、下列词汇**网络语言**当中比较流行的词汇，请您根据自己对它们的熟悉程度在其后的数字上打√。

		完全没听过	听过但不知道明确的意思	知道意思但没用过	偶尔使用过	使用的情况一般（不太频繁）	经常使用（很频繁）
1	Hold 住	①	②	③	④	⑤	⑥
2	土豪	①	②	③	④	⑤	⑥
3	悲催	①	②	③	④	⑤	⑥
4	躺枪	①	②	③	④	⑤	⑥
5	菜鸟	①	②	③	④	⑤	⑥
6	杯具	①	②	③	④	⑤	⑥
7	吃货	①	②	③	④	⑤	⑥
8	坑爹	①	②	③	④	⑤	⑥
9	逆袭	①	②	③	④	⑤	⑥
10	脑残	①	②	③	④	⑤	⑥
11	秒杀	①	②	③	④	⑤	⑥
12	萝莉	①	②	③	④	⑤	⑥
13	逆天	①	②	③	④	⑤	⑥
14	奇葩	①	②	③	④	⑤	⑥
15	小强	①	②	③	④	⑤	⑥

16	节奏	①	②	③	④	⑤	⑥
17	脑残粉	①	②	③	④	⑤	⑥
18	矮穷挫	①	②	③	④	⑤	⑥
19	女汉子	①	②	③	④	⑤	⑥
20	打酱油	①	②	③	④	⑤	⑥
21	有木有	①	②	③	④	⑤	⑥
22	我(勒个)去	①	②	③	④	⑤	⑥
23	你懂的	①	②	③	④	⑤	⑥

Supplementary materials 2：

Word writing test

1、写字部分（该部分已经给出词语其中一个字，请写出另一个字）

ēn huì 恩______	lā sà 拉______	yào shi 钥______	juàn dài 倦______	zhì xī______息
diāo xiè______谢	tōu qiè 偷______	kè zhàn 客______	chèn yī ______衣	jiě pōu 解______
hé xié 和______	yù mèn______闷	shěnglüè 省______	dù jì ______忌	guǐ jì ______计
là jiāo______椒	fú lǔ 俘______	tiǎo xìn 挑______	xíng huì 行______	chà dào______道
zhuózhuàng______壮	lún dūn 伦______	shē qiàn______欠	pò zhàn 破______	shē chǐ______侈
yú lè______乐	zhù zhái 住______	tuǒ xié______协	jǔ sàng______丧	kuàng yě______野
nǎi lào 奶______	xiàn mù______慕	nüè dài______待	kū jié 枯______	mián xù 棉______
qián é 前______	qiān míng______名	xiá gǔ______谷	bèi pàn 背______	jiāo wài______外

2、写词部分（该部分词语已标出拼音，请填出正确的词，以使句子意义完整）

（1）我这个外出的游子经常会思念 **cí xiáng**（　　　　）的母亲。

（2）他的脸上 **yáng yì**（　　　　）着会心的笑容。

（3）**qí qū**（　　　　）的山路一直弯弯曲曲通向山顶。

（4）河边有个穿着 **suō yī**（　　　　）的老翁在小雨中垂钓。

（5）这个城市是通往东西南北的交通 **shū niǔ**（　　　　）。

（6）爬过这座土山，我们看到了一望 **wú yín**（　　　　）的沙漠。

（7）他有事情 **dān ge**（　　　　）了，可能要迟一点。

（8）身上绑着橡皮绳，从几十米的高度往下跳，**bèng jí**（　　　　）是勇敢者的运动。

（9）这是一片 **pín jí**（　　　　）的土地，几乎什么也不长。

（10）他擅长水墨 **dān qīng**（　　　　），是绘画大师。

（11）每到黄昏的时候，小镇上家家户户屋顶上就会升起 **niǎo niǎo**（　　　　）炊烟。

（12）她穿一身迷彩服，显得英姿 **sà shuǎng**（　　　　）。

（13）今天是商场开业打折的日子，购物的人们 **fēng yōng**（　　　　）而来。

（14）这个碗是 **táo cí**（　　　　）制的。

（15）这个故事明显是 **dù zhuàn**（　　　　）的，历史上没有这个人。

（16）听到别人对自己的议论，她感觉犹如 **máng cì**（　　　　　）在背。

（17）等他们赶到时，只发现一堆动物尸体的 **cán hái**（　　　　　）。

（18）他因为劳累而倒在工作岗位上，是因公 **xùn zhí**（　　　　　）。

（19）山西是产 **méi tàn**（　　　　　）的大省。

（20）当演讲者表明他的观点后，全场 **yǎ rán**（　　　　　），一片寂静。

（21）他喜欢激烈运动，出一身大汗，让他感觉 **hān chàng**（　　　　　）淋漓。

（22）警察找到了凶器，那是一把 **bǐ shǒu**（　　　　　）。

（23）初稿比较潦草，于是我又认真 **téng xiě**（　　　　　）了一遍。

（24）大师是出家人，饮食不沾 **hūn xīng**（　　　　　），只吃素食。

（25）经过几百里地的长途 **bá shè**（　　　　　），他们终于到达了目的地。

（26）我国古代有 **hóng yàn**（　　　　　）传书一说。

（27）我一定会履行我的 **chéng nuò**（　　　　　）。

（28）宝剑锋从 **mó lì**（　　　　　）出，梅花香自苦寒来。

（29）这栋大楼，建了两年终于 **jùn gōng**（　　　　　）了。

（30）在这个世界有些贫穷的地方，人们依旧食不 **guǒ fù**（　　　　　），衣不遮体。
